# Paradoxical, causal effects of sensory gain modulation on motor inhibitory control – a tDCS, EEG-source localization study

**DOI:** 10.1038/s41598-018-35879-2

**Published:** 2018-11-30

**Authors:** Julia Friedrich, Christian Beste

**Affiliations:** Cognitive Neurophysiology, Department of Child and Adolescent Psychiatry, Faculty of Medicine, TU Dresden, Germany

## Abstract

Response inhibition is a key component of executive functioning, but the role of perceptual processes has only recently been focused. Although the interrelation of incoming information and resulting behavioural (motor) effects is well-known to depend on gain control mechanisms, the causal role of sensory gain modulation for response inhibition is elusive. We investigate it using a somatosensory response inhibition (Go/Nogo) task and examine the effects of parietal (somatosensory) cathodal and sham tDCS stimulation on a behavioural and neurophysiological level. For the latter, we combine event-related potential (ERP) and source localization analyses. Behavioural results reveal that cathodal stimulation leads to superior inhibition performance as compared to sham stimulation depending on the intensity of tDCS stimulation. The neurophysiological data show that an early (perceptual) subprocess of the Nogo-N2 ERP-component is differentially modulated by the type of stimulation but not a later (response-related) Nogo-N2 subcomponent. Under cathodal stimulation, the early N2 amplitude is reduced and the right inferior frontal gyrus (BA45) is less active. Cathodal tDCS likely enhances inhibition performance via decreasing the efficiency of gain control and the impact of sensory stimuli to trigger prepotent responses. Thereby, response inhibition processes, associated with structures of the response inhibition network, become less demanded.

## Introduction

In everyday life, sensory information is constantly used to guide behaviour. Short-term changes of the environment sometimes require “deliberate overriding of dominant or prepotent responses”^1^ which describes one of the core executive functions, namely response inhibition. In the last years, the role of perceptual processes during response inhibition has attracted a considerable interest^2–4^ and it has been shown that the somatosensory modality is very potent to trigger response inhibition^4–7^. One reason is the strong structural anatomical connection between the parietal somatosensory cortices (i.e. the primary (SI) and secondary somatosensory cortex (SII)) and the posterior parietal cortex (PPC) with the motor system^8^ and prefrontal brain areas implicated in response inhibition processes^9–11^. However, on a neuronal level, the interrelation of a bottom-up sensory input processing to its effect (output) is subject to gain control mechanisms^12–15^. When gain is high, the impact of input on output activations is enhanced. The opposite is the case when gain control is lowered^12–18^. Although the interrelation of incoming information and resulting effects is well known to depend on gain control mechanisms, the causal role of sensory gain modulation for response inhibition is elusive.

We propose that reduced gain control may not always compromise behavioural output and we hypothesize that it may rather enhance inhibitory control. This is because the neuronal representation of the GO stimulus strongly affects the strength of the evoked prepotent response tendency^19–21^. If the neuronal representation of GO stimuli is attenuated by reduced neuronal responsivity of the processing brain area, GO trials may be less potent to trigger response tendency. As a consequence, it is easier to inhibit the response and response inhibition processes become more effective.

One possibility to address this hypothesis is the application of transcranial direct current stimulation (tDCS). The reason is that gain control is directly affected by neuronal excitability; i.e. when membrane potentials are high, weaker inputs can trigger neuronal responses. On the contrary, decreased levels of membrane potentials reduce input sensitivity and thus gain control^16,22,23^. TDCS modulates cortical excitability through the subthreshold modulation of membrane potentials and the probability of spontaneous neuronal activity is decreased by cathodal stimulation^24–27^. Moreover, tDCS has recently been shown to affect the neuronal input/output function and the likelihood of neuronal firing for a given and fixed synaptic input^28^; i.e. properties that have been conceptualized as gain modulation. The processes likely affect the strength of the neuronal representation of stimuli. It is therefore possible to examine whether (somato)sensory gain control mechanisms are causally involved in the modulation of motor inhibition using tDCS. We investigate this in a tactile GO/NOGO task^6^ and examine associated neurophysiological mechanisms using EEG recordings, i.e. event-related potential (ERP) and source localization (sLORETA) analyses. We focus on the effects of cathodal stimulation, since this has been shown to produce large and consistent effects^29,30^. On the ERP level, the Nogo-N2 is assumed to mirror pre-motor processes like detection or monitoring of conflict or the updating of the required response, respectively^31–34^. The P3, however, more likely reflects later response evaluation-associated processes^32,35,36^ or the motor inhibition per se^37–39^. Importantly, it has been shown that the (Nogo)-N2 ERP correlate reflects concomitant signals of perceptual and cognitive control and/or response selection processes^40,41^. The N2 should therefore be considered as a composition of subcomponents reflecting different processes: one subcomponent that is rather modulated by perceptual processes and another subcomponent more closely linked to cognitive control^40^. Since gain control processes relate to the modulation of sensory information processing, it is likely that the (Nogo)-N2 component reflects modulations on the perceptual rather than on the cognitive control level. This is because cathodal stimulation is assumed to reduce neuronal excitability by a decrease in membrane potential. Therefore, it is hypothesized that experimental manipulation in this study will cause a “dissociation” of the N2 in the way that the type of stimulation (sham/cathodal) modulates N2 subprocesses likely reflecting perceptual processing and not the subprocesses presumably reflecting cognitive, or pre-motor control. We assume that a cathodal tDCS induced reduction in excitability also reduces gain control by decreasing signal-to-noise ratio. When the signal-to-noise ratio is attenuated, the strength of neuronal representation of the GO stimulus is expected to decline. This is assumed to reduce the prepotent response tendency associated with the GO stimulus so that inhibition becomes more effective; i.e. the “braking function” of inhibitory control is more efficient. Previous research suggests that this “braking function” is associated with processes in the right inferior frontal gyrus (rIFG)^42–45^. It is therefore possible that even though tDCS is applied above the somatosensory cortex to modulate sensory gain control mechanisms, processing in frontal areas and parts of the response inhibition are affected. However, it cannot be excluded that effects also reach the response selection level. Therefore, also later occurring components (i.e. P3) are analyzed.

## Results

### Behavioural data

The paired t-test conducted for the GO hit rate revealed no significant difference between sham and cathodal stimulation [*t*(16) = 1.76; *p* = 0.097]. Examination of hit reaction times showed a similar pattern with no significant effect of the factor “stimulation type” [*t*(16) = −0.36; *p* = 0.723]. The paired t-test conducted for the false alarm rate in NOGO trials demonstrated that the “stimulation type” had a significant effect [*t*(16) = −2.43; *p* = 0.027]. More errors occurred in the sham (6.8% ± 1.3) than in the cathodal stimulation condition (5.1% ± 1).

### Event-related potentials (ERPs)

**Figure 1:**
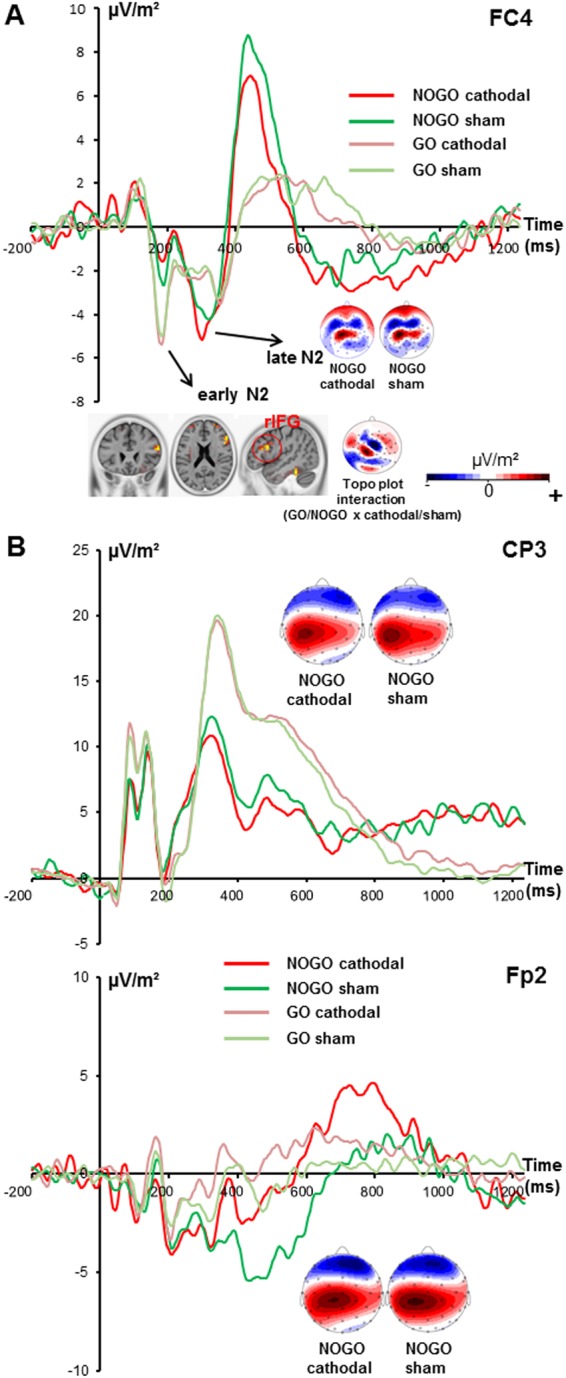
The P3 and N2 ERP-components at electrodes FC4 (upper part **A**), CP3 and Fp2 (lower part **B**). Please note the different y-axes scaling for better illustration of neurophysiological time course at different electrodes. Time point 0 represents stimulus presentation. The different colors of the ERPs reflect the cathodal and sham stimulation condition in GO and NOGO trials as can be seen in the legend. The scalp topography plots represent the N2 and P3 in NOGO trials for the different conditions with red indicating positive and blue negative values. The sLORETA plots indicate the source of the difference in early N2 amplitude modulations in NOGO trials in the sham and cathodal stimulation condition. The respective color scale presents critical t-values (corrected for multiple comparisons using SnPM).

The ERP data are shown in Fig. 1 at electrode FC4 in GO and NOGO trials in the sham and cathodal tDCS condition.

As can be seen in Fig. 1, the potentials in the N2 time range split in two peaks: one peak in the time interval from 160 to 180 ms and another peak between 275 to 290 ms. For the first peak of the N2 between 160 and 180 ms after stimulus presentation (henceforth called “early N2”), the mixed effects ANOVA of the N2 revealed a main effect of „trial type“ [F(1,16) = 9.44; *p* = 0.007; ${\eta }_{p}^{2}$ = 0.371] showing that the amplitude was larger (i.e. more negative) in GO (−4.59 μV/m² ± 1.48) than in NOGO trials (−1.87 μV/m² ± 0.89). Furthermore, the interaction of “stimulation type x trial type” was significant [F(1,16) = 5.37; *p* = 0.034; ${\eta }_{p}^{2}$ = 0.251]. A power analysis shows that having a sample of N = 17 subjects and an obtained effect size in the critical interaction of ${\eta }_{p}^{2}$ = 0.251, the achieved power is larger than 95%. Therefore, the sample size is sufficiently large to derive reliable conclusions. Post-hoc paired t-tests revealed that there was no significant difference between the stimulation types in GO trials [*t*(16) = 0.19; *p* = 0.853] but in NOGO trials [*t*(16) = −2.00; *p* = 0.032]. In NOGO trials, the sham stimulation resulted in a larger early N2 amplitude (−2.52 μV/m² ± 1.03) than the cathodal stimulation (−1.22 μV/m² ± 0.87). To examine which functional neuroanatomical sources underlie these modulations in NOGO trials, sLORETA was applied. As can be seen in Fig. 1, there was more activation in the rIFG (BA 45) in the sham than in the cathodal stimulation condition. The main effect of “stimulation type” was not significant [F(1,16) = 0.91; *p* = 0.353; ${\eta }_{p}^{2}$ = 0.054]. For the second peak in the N2 time window between 275 and 290 ms (henceforth called “late N2”), the mixed effects ANOVA showed a main effect of “trial type” [F(1,16) = 8.33; *p* = 0.011; ${\eta }_{p}^{2}$ = 0.342] with a larger amplitude in NOGO (−4.54 μV/m² ± 1.55) than in GO trials (−2.09 μV/m² ± 1.33). All other main or interaction effects failed to reach significance [all F ≤ 2.72; *p* ≥ 0.118]. As can further be seen in Fig. 1, a Nogo-P3 component was evident as well. Because the “late N2” just before the Nogo-P3 revealed amplitude modulations depending on trials type, peak-to-peak amplitudes were used for the analysis of the Nogo-P3. The mixed effects ANOVA for peak-to-peak P3 comparison revealed a significant main effect of “trial type” [F(1,16) = 26.01;; *p* < 0.001; ${\eta }_{p}^{2}$ = 0.619] with a larger (more positive) amplitude in NOGO trials (12.03 μV/m² ± 2.07) than in GO trials (3.37 μV/m² ± 1.45). All other main or interaction effects failed to reach significance [all F ≤ 2.05; *p* ≥ 0.657].

Mixed effects ANOVA for the early N2 at electrode CP3 revealed no significant main or interaction effects [all F ≤ 2.6; *p* ≥ 0.126]. For the late N2 time window, a significant main effect of “trial type” was evident [F(1,16) = 5.34;; *p* = 0.035; ${\eta }_{p}^{2}$ = 0.250] revealing a more positive amplitude in NOGO (5.34 μV/m² ± 1.29) than in GO trials (2.01 μV/m² ± 1.9). Regarding the lack of negative values when quantifying the late N2 it becomes obvious, that early and late N2 processes are not evident at electrode CP3 but are illustrated at electrode FC4. No other main or interaction effects were significant [all F ≤ 0.04; *p* ≥ 0.842]. For P3 ERP the mixed effects ANOVA showed a main effect of “trial type” [F(1,16) = 23.4; *p* < 0.001; ${\eta }_{p}^{2}$ = 0.594] with larger amplitudes in GO 17.65 μV/m² ± 2.06) than in NOGO trials (11.34 μV/m² ± 1.38). No further main or interaction effects were significant [all F ≤ 0.69; *p* ≥ 0.419].

For the early N2, mixed effects ANOVA for electrode Fp2 revealed no significant main or interaction effect [all F ≤ 3.43; *p* ≥ 0.083]. Also for the late N2, no significant main or interaction effects occurred [all F ≤ 1.21; *p* ≥ 0.288]. For the P3 time window, no significant main or interaction effects were shown [all F ≤ 2.48; *p* ≥ 0.135].

In addition to the 2 mA cathodal tDCS stimulation, we examined the effects of 1 mA cathodal stimulation on a behavioural level (i.e. no EEG recordings were performed). This was done because it cannot be excluded that gain control mechanisms have to exceed a certain threshold to effectively modulate response inhibition. We tested N = 16 participants (8 females) between 18 and 30 years (mean age = 23; SEM = 0.97). The paired t-test analyzing GO hit rate resulted in no significant difference between sham and cathodal stimulation [*t*(15) = −1.31; *p* = 0.209]. Furthermore, the paired t-test conducted for the effect of stimulation on hit reaction times was also not significant [*t*(15) = 0.73; *p* = 0.480]. Also the paired t-test calculated for the false alarm rate in NOGO trials revealed no significant effect of stimulation types [*t*(15) = 0.32; *p* = 0.750].

## Discussion

This study was conducted to examine the causal relevance of gain control mechanisms modulating sensory information processing for motor inhibitory control. This was done using a tactile GO/NOGO paradigm^6^. To examine the underlying system neurophysiological mechanisms modulated by tDCS-induced gain control mechanisms, ERP and source localization analyses (sLORETA) were combined. The results show that response inhibition performance was better in the cathodal stimulation condition than the sham stimulation condition, which is reflected by the significantly higher false alarm rate in the sham condition. Cathodal tDCS has been shown to reduce the cortical excitability of the somatosensory cortex^29^ and decreases the level of membrane potentials^24^. This decline (modulation) in neuronal excitability acts as a kind of gain control mechanism reducing the responsivity to neuronal input resulting in a decreased signal-to-noise ratio and a weakening of the neuronal stimulus representation^13–15,28^. The results from the experiment using 1 mA cathodal tDCS stimulation demonstrating no significant behavioural effect, however suggest that the reduction of neuronal responsivity has to reach a certain threshold to effectively modulate response inhibition.

In the paradigm, GO trials were more frequent than NOGO trials, which leads to strong representation of GO stimuli and induces a prepotent response tendency^20,46–48^. The attenuation of gain control mechanism by means of cathodal tDCS likely weakens the neuronal responsivity to external input and hence also the impact of GO stimuli. Therefore, the prepotent response tendency may also be reduced making it easier to inhibit the response. This means that although attenuated gain control has frequently been associated with impaired performance^13^, behavioural inhibition can improve. It may be argued that cathodal tDCS effect will also affect processing of NOGO stimuli. Yet, if both trial types were affected in the same manner, there would have been more false alarms in the cathodal than in the sham condition. The reason is as follows: When NOGO representations are attenuated, response inhibition is less effectively triggered, which increases false alarm rates. Since results revealed the opposite pattern, cathodal tDCS effects seem to be confined to the representation of GO trials.

The neurophysiological data provide more information about mechanisms underlying these effects. It was hypothesized that tDCS-induced changes in gain control mechanisms affect perceptual aspects of stimulus processing and less so cognitive or pre-motor aspects of inhibition. Importantly, several lines of evidence suggest that the N2 reflects concomitant signals of perceptual and cognitive control and/or response selection processes^40,41^. In fact, the N2 seems to reveal different subprocesses in the current study that are also differentially affected by tDCS: For the “early N2” time window between 160 to 180 ms an interaction of trial (GO/NOGO) and stimulation type (cathodal/sham) paralleling the behavioural result was found. This was not the case for the “late N2” time window between 275 to 290 ms. Likely, the “early N2” (between 160 to 180 ms) may reflect perceptual subprocesses of the N2^40^. The “late N2” revealed the usual pattern of a larger amplitude in NOGO than in GO trials and a usual latency^49–51^. We therefore interpret the „late N2“ to reflect the usual Nogo-N2, which has been suggested to reflect pre-motor aspects of inhibition^32^. Regarding the “early N2” it may be confusing that the amplitude is more negative in GO than in NOGO trials. Actually, this pattern seems to depict a clear difference between the visual and the tactile system, which has already been demonstrated^5,6^ thus making an incidental finding unlikely. One can only speculate that the tactile modality is more effectively triggering the prepotent GO response tendency due to strong neuronal connections of somatosensory and frontal motor areas^9,52,53^ so that the inhibition system is constantly “on alert” in GO trials to avoid a premature response. Therefore, it is possible that response tendency is permanently inhibited leading to an increased N2 in GO trials to prevent premature responding in a sudden NOGO case. Most importantly, in NOGO trials the cathodal stimulation resulted in a smaller “early N2” amplitude than the sham stimulation condition. Therefore, better inhibition performance was associated with a smaller “early N2” amplitude. The reason might be as follows: cathodal tDCS decreases membrane potentials in neural circuits. This decreases the likelihood that the neural circuit is activated and reduces the potentials evoked by the sensory input. This reduces the strength of neuronal GO stimulus representation and causes a diminution of prepotent response tendency, which therefore also reduces the conflict assumed to be reflected by the N2^33^. As a consequence it becomes easier to inhibit a motor response. This line of arguments may seem extraordinary since a larger N2 amplitude usually reflects stronger response inhibition and hence better inhibition performance^40,54^. This observation might be again pointing to a significant difference between the visual and tactile domain^5,6^. Since the neuronal GO representation is attenuated by cathodal tDCS (see above) the above mentioned constant “alert mode” of the inhibitory system in order to avoid premature responding in a sudden NOGO case is reduced also decreasing the strength of inhibitory processes needed. Thus, results on the early perceptual N2 suggest that effects on response inhibition might be driven by gain modulation of sensory information. This might also explain why no Nogo-P3 amplitude modulations were evident, because the Nogo-P3 frequently been associated with the motor inhibition per se^39^ and/or response evaluation processes^32^; i.e. mechanisms unrelated to sensory information processing. Results fit into existing literature describing an advantage of active stimulation for inhibitory control in different settings^55–57^. Nevertheless, integrating the study into other work in the field of inhibitory control is difficult since stimulation sites, applied paradigms and examined modalities broadly differ. To our knowledge, it is the first study investigating the effect of somatosensory modulation on motor inhibitory control by means of a tactile GO/NOGO paradigm.

Interestingly, amplitude modulations of the early N2 were linked to activation differences in the right inferior frontal gyrus (rIFG) as reflected by sLORETA results. This region has frequently been shown to be involved in response inhibition^32,58,59^. This suggests that even though tDCS was applied over parietal regions, gain control modulations have a “long-range” effect on prefrontal areas. It has already been shown that functional connectivity between remote areas is changed by tDCS^60,61^ making it plausible that remote effects of tDCS are evident in the current data. The rIFG has been suggested to be part of a response inhibition network^44,45^ and likely subserves a “braking function”^42–45^. Since the response inhibition network usually comprises right lateralized parieto-frontal areas^62^, it is not surprising that neurophysiological effects were evident at electrode FC4. Interestingly, there are strong structural anatomical connections between the somatosensory cortex and prefrontal areas including the rIFG^9,52,53,63^ which are also assumed to underlie the high efficiency of somatosensory stimuli to trigger response inhibition processes^5,6^. It is possible that the remote effects of parietal tDCS and gain modulations processes on motor inhibitory control emerge due to these neuroanatomical connections between prefrontal and parietal regions.

In summary, the study shows that response inhibition is causally affected by sensory gain modulation. Cathodal tDCS likely enhances inhibition performance via decreasing the efficiency of gain control and the impact of sensory stimuli to trigger prepotent responses. Thereby, response inhibition processes, associated with structures of the response inhibition network, become more efficient. As a future perspective, it should be considered to validate the core finding of this study that cathodal tDCS enhances inhibition performance by investigating clinical populations that are characterized by inhibitory response disruption like Parkinson’s or Huntington’s disease^35,64^ or attention deficit hyperactivity disorder (ADHD) population^65,66^.

## Materials and Methods

### Participants

N = 17 subjects participated in this experiment. The age range was between 19 and 29 years (mean age = 23; SEM = 0.65) and they all confirmed to be right-handed. A written informed consent was signed by the participants. They reported no psychiatric or neurological disorders and were checked for tDCS eligibility. All subjects provided written informed consent in accordance with the Helsinki Declaration of 1975, as revised in 2008. All methods were performed in accordance with the relevant guidelines and regulations. The study was approved by the local ethics committee of the Medical Faculty of the TU Dresden.

### Procedure and task

**Figure 2:**
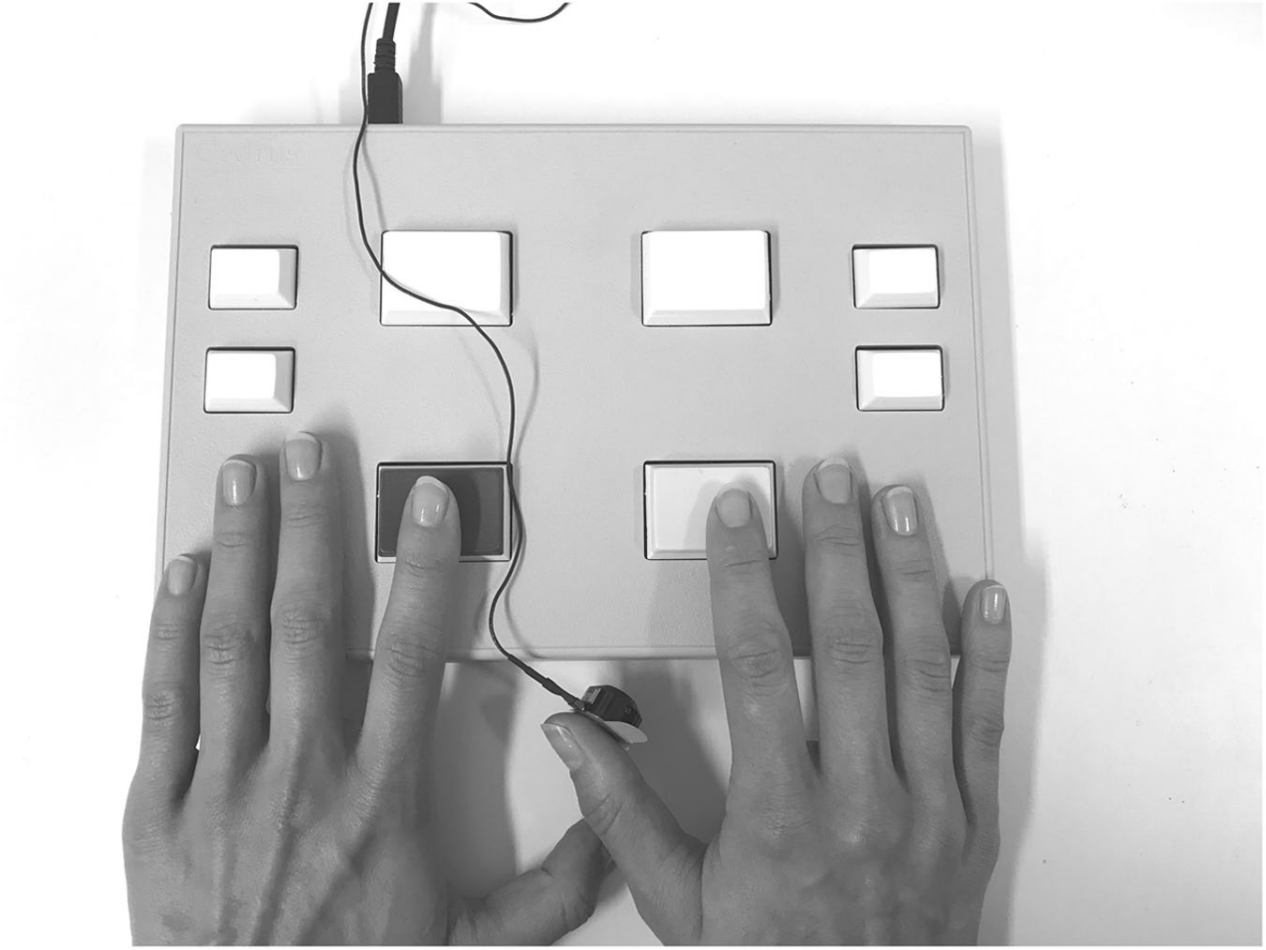
Illustration of the experimental setup. The stimulator was attached to the right thumb to prevent contact with the table and the response device. Participants were told to respond by button press with their right or left index finger depending on the current condition (left/right hand response).

In two separate sessions, participants either received cathodal or sham stimulation prior to the experiment. The sequence of the type of tDCS stimulation was counterbalanced across participants. First, participants received the stimulation and afterwards the EEG cap was placed and prepared. Subsequently, the task was performed. We applied cathodal tDCS predominantly targeting the somatosensory cortex to modulate the processing of tactile information. This was done to investigate the effect this modulation has on the execution of motor inhibitory processes. Therefore, we conducted a GO/NOGO task with vibro-tactile stimuli^6,7^. Small electromagnetic stimulators (Dancer Design; for more detailed information see http://www.dancerdesign.co.uk controlled by a “main module” (Neurocore; http://www.neurocore.de/) were used to generate vibro-tactile stimuli. The experimental setup is shown in Fig. 2.

GO and NOGO stimuli had different frequencies: A 40 Hz frequency constituted the NOGO stimulus and the 150 Hz stimulation the GO stimulus. Stimulation frequencies were chosen based on previous results of our lab demonstrating that 40 Hz vibro-tactile stimulation predominantly processed in the SI cortex^67–69^ is effectively modulating response inhibition^6^. The amplitude of both frequencies was equal and stimulation duration was set to 100 ms for both types of stimuli in order to evoke a strong response tendency so that inhibitory processes are triggered in NOGO trials. This short period of time was chosen to reduce the time participants have for stimulus categorization to increase the probability for premature responses to occur. Two types of responses were required from each subject. The subjects either had to press the right button with their right index finger or the left button with their left index finger when the GO stimulus was presented whereas no response was required in response to the NOGO stimulus. These two types of required responses were arranged in blocks so participants would not have to switch between different responses within a block. This manipulation was implemented to investigate whether solely motor inhibitory processes required to withhold the right hand response are affected or whether the unilateral stimulation influences both effectors due to transcallosal connections^9,70^. The block sequence was either ABBA or BAAB with A and B representing blocks requiring either a left or right hand response in order to reduce the anticipation of a certain block. Prior to each block, participants received the information whether a left or a right index finger response was required. Block sequence was counterbalanced across stimulation conditions. In sum, 4 blocks comprising a total of 832 trials were presented. The trial sequence within the blocks was pseudorandomized and trial onset was jittered from 700 to 1100 ms to avoid an anticipation of stimulus appearance. 70% of trials were GO trials and 30% NOGO trials. In case the response button was pressed within 100 to 1000 ms after GO stimulus presentation, the answer was classified as correct. In NOGO trials no response should occur in the same time period. Each subject accomplished two left hand and two right hand response blocks.

### EEG recording and analysis

EEG recording was conducted using 60 passive Ag/AgCl ring electrodes at equidistant positions connected to a QuickAmp amplifier (BrainProducts Inc.). Ground and reference electrodes were positioned at coordinates theta = 58, phi = 78 and theta = 90, phi = 90, respectively. Data was sampled with a rate of 500 Hz and the recording bandwidth ranged from 0.5 to 80 Hz. Afterwards, a down-sampling to 256 Hz was conducted and the un-epoched data set was filtered from 0.5 Hz to 20 Hz with a slope of 48db/oct applying an IIR band-pass filter. Furthermore, a 50 Hz notch filter was used. During the first manual raw data inspection infrequent (technical or muscular) artifacts were removed. To identify artifacts originating from eye movement like blinks or lateral movements and pulse artifacts, an independent component analysis was conducted (ICA; infomax algorithm) for all blocks combined. A manual rejection of ICA components reflecting artifacts followed.

Then, GO and NOGO trials were segmented separately for the sham and the cathodal stimulation condition. A segment started −200 ms before and ended 1200 ms after stimulus presentation. Solely trials that were answered correctly were included in analyses. Correct trials were defined as pressing the response button in GO trials between 100 ms to 1000 ms after stimulus presentation and to refrain from responding in the same interval in NOGO trials. Subsequently, an automated artifact rejection procedure eliminated trials with a maximal value difference of 200 μV in a 200 ms time interval as well as trials with amplitudes below −200 μV and above 200 μV and below 0.5 μV in a 100 ms interval. Subsequently, current source density (CSD) transformation was used to eliminate the reference potential from the data and to re-reference the data resulting in amplitude values in μV/m^2^. We used 4 splines and 10 polynominals. CSDs work as a spatial filter^71,72^, which accentuates electrode sites and makes it easier to identify electrode sites that best reflect relevant neuronal activity. Afterwards, data was baseline corrected from −200 to 0 (with 0 representing stimulus presentation). Then, the average of the different conditions was first formed for each subject separately and subsequently grand averages were calculated for GOs and NOGOs for the real and sham stimulation condition.

Based on the visual inspection of scalp distributions and averaged EEG data courses components and electrodes of interest were preselected. To validate and refine this choice difference waves between the cathodal and sham stimulation condition were calculated for both trial types. N2 and P3 ERPs were most clearly shown at electrode FC4. The N2 was quantified in several time intervals on the basis of the mean amplitude to best depict effects reflected by this ERP. Calculations were conducted for both trial (GO/NOGO) and stimulation types (sham/cathodal) separately. An early N2 was calculated for the time range between 160 to 180 ms. A late N2 was quantified in the time interval from 275 to 290 ms. To cover the intermediate period between the early and the late N2 in which the underlying process showed an altered course, the interval from 195 to 215 ms was also quantified. Furthermore, a peak-to-peak quantification for the P3 was conducted. The mean amplitude in the time interval between 275 to 290 ms was therefore subtracted from the mean amplitude in the time interval between 400 to 440 ms for both trial and stimulation types (i.e. GO and NOGO trials in the sham and cathodal condition, respectively). Furthermore, early and late N2 as well as P3 ERP was quantified at electrodes CP3 to see whether tDCS has proximal effects at stimulation location. For early N2 the time window for 160 to 180 ms was used and for late N2 the time window between 210 to 240 ms. P3 ERP was quantified in the time range between 285 to 320 ms. Additionally, analyses were conducted for electrode Fp2 to examine whether the orbitofrontal cortex might contribute to observed effects. Early N2 was quantified in the time window between 160 and 180 ms and late N2 in the range between 275 and 290 ms. P3 ERP was calculated for the time range between 305 and 340 ms.

### Source localization analysis

For the source localization analysis, sLORETA (standardized low resolution brain electromagnetic tomography)^73^ was used. The source localization analysis was based on the early N2 ERP component reflecting the behavioral interaction. sLORETA provides a single solution to the inverse problem^73–75^. Therefore, the intracerebral volume is partitioned into 6239 voxels at 5 mm spatial resolution. Subsequently, the standardized current density at each voxel is calculated in a realistic head model^76^ based on the MNI152 template^77^. There is mathematically substantiated evidence that sLORETA provides reliable results without a localization bias^75^. Additionally, there is evidence from EEG/fMRI and neuronavigated EEG/TMS studies validating the sources estimated using sLORETA^75,78^. The voxelbased sLORETA images were compared across conditions (NOGO trials in the cathodal vs. sham stimulation condition) using the sLORETA-built-in voxel-wise randomization tests with 2000 permutations, based on statistical nonparametric mapping (SnPM). Voxels reflecting significant differences (p < 0.01, corrected for multiple comparisons) between contrasted conditions were located in the MNI-brain.

### Transcranial direct current stimulation

**Figure 3:**
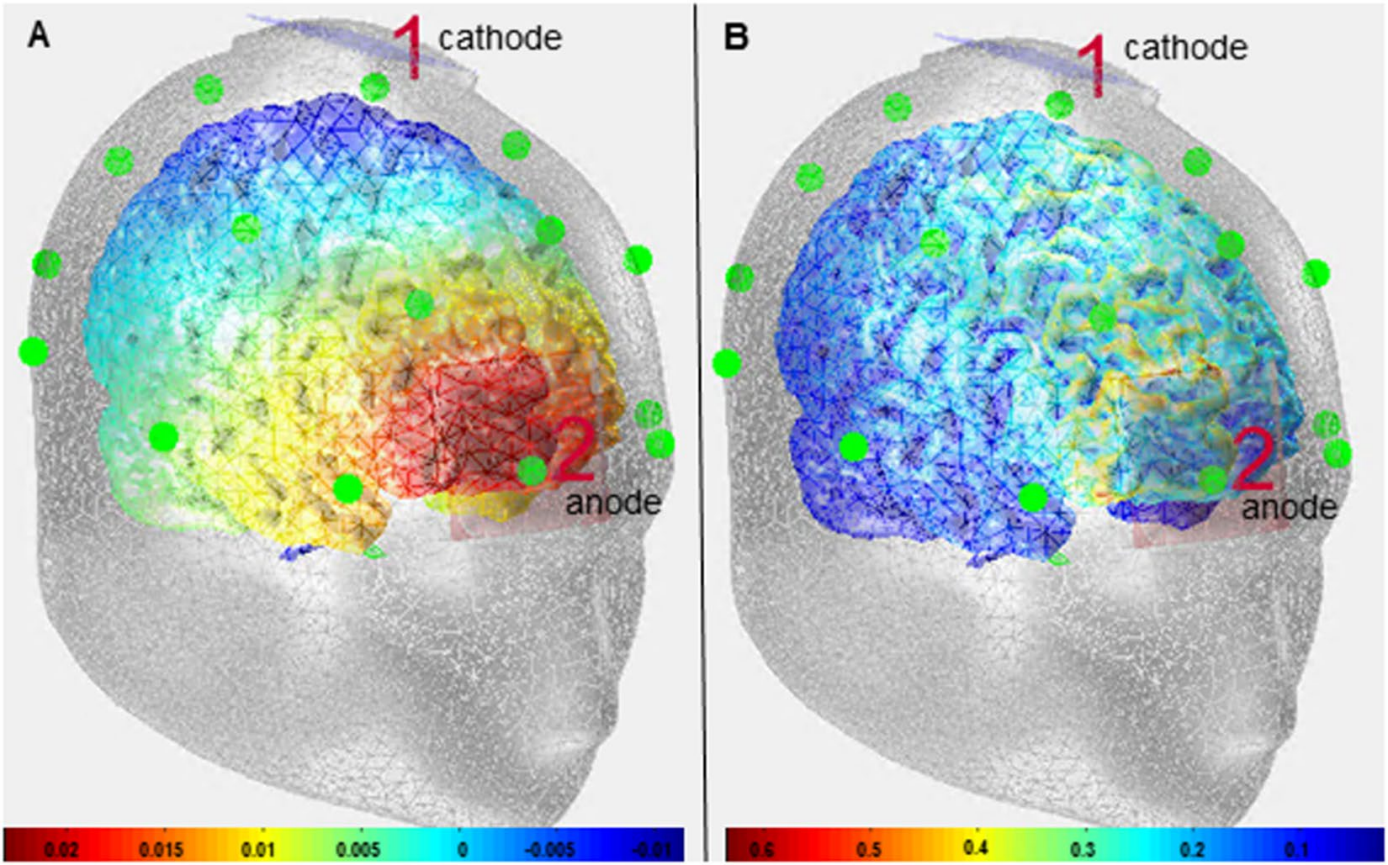
Simulation of electrical current flow via Comets Toolbox (MATLAB 12.0; Mathworks Inc.). Electric potential in Volt (left part **A**). Current density in Joule (right part **B**).

A battery-driven stimulator (DC-Stimulator Plus, NeuroConn, Illmenau, Germany) was used to induce the 2 mA current delivered via two rubber electrodes (5 × 5 cm², NeuroConn, Illmenau, Germany) fixated with two elastic straps. The cathode was centered over electrode CP3 in an equidistant system covering the commonly described position used to target the somatosensory cortex (2 cm posterior to C3 electrode)^79–82^. The anode constituting the reference electrode was placed above the contralateral orbit roughly parallel to the eyebrow^83^. To simulate the current flow of this electrode montage (Fig. 3), we used the Comets Toolbox (MATLAB 12.0; Mathworks Inc.). As can be seen in Fig. 3 the main focus of stimulation is above somatosensory and orbitofrontal/frontopolar regions. This reference placement was used to guarantee adequate distance between both electrodes to prevent the current from being shunted through the skin^84^. Furthermore, the orbitofrontal cortex is not considered to be involved in motor inhibitory control processes^85,86^. Thus, its anodal stimulation is not assumed to modulate response inhibition. To avoid direct contact of the electrodes and the scalp and to ensure that the current is distributing homogenously, Ten20 conductance paste (0.5 mm thick) was used to attach the electrodes^27^. Constant thickness of the paste was guaranteed by using a special plastic frame for preparation. Impedances were below 5 kΩ. The current was faded in over a period of 10 seconds^80,82,87^ to minimize unpleasant sensations^25,88,89^. Fading out was set to 30 seconds to further reduce sensations and hence the perceived contrast between cathodal and sham stimulation. The DC-stimulator was installed and controlled outside of the view of the participants so that they were blind in regard to the current condition. In the sham condition, current was delivered for 30 seconds^81,82,87,90^. Active stimulation was adjusted to 900 seconds in order to produce aftereffects on the excitability of the somatosensory cortex outlasting up to 60 minutes^29^ and therefore affecting task performance. After stimulation two experimenters prepared the EEG cap which lasted approximately 15 minutes. While the cap was placed and prepared, the participants filled in a questionnaire. Subsequently, the task (lasting approximately 20 minutes) was performed so the task performance was accomplished within the 60 minutes described above. To produce a sufficiently strong modulation of the somatosensory cortex that is also affecting the response inhibition process the stimulation duration and intensity was slightly increased to 2 mA still complying with tDCS safety criteria^89,91,92^. Cathodal stimulation was conducted since it frequently resulted in larger effects as opposed to anodal stimulation^29,30,83,93^ as it was also shown for the visual domain^94^. Mean time delay between both sessions was 9.1 days (SEM = 0.86).

### Statistical analysis

Paired t-tests were used to analyze behavioural data (i.e. percentage of hits in GO trials as well as hit reaction times and the false alarm rates in NOGO trials). Therefore the two levels of the factor “stimulation type” (cathodal/sham) were compared. Mixed effects ANOVAs were conducted for neurophysiological data using the within-subject factors “trial type” (GO/NOGO) and “stimulation type”. The factor “response hand” (left/right hand response) was excluded from analyses since there was no differential effect of stimulation type [F(1,16) = 0.29; *p* = 0.600; ${\eta }_{p}^{2}$ = 0.018]. Greenhouse-Geisser correction was applied to all tests and Bonferroni correction to all post hoc tests. Below, mean values and the standard error of the mean (SEM) were put in brackets illustrating descriptive statistics.

## Data Availability

The datasets generated during and/or analysed during the current study are available from the corresponding author on reasonable request.
